# Evaluation of a Web Portal for Improving Public Access to Evidence-Based Health Information and Health Literacy Skills: A Pragmatic Trial

**DOI:** 10.1371/journal.pone.0037715

**Published:** 2012-05-31

**Authors:** Astrid Austvoll-Dahlgren, Arild Bjørndal, Jan Odgaard-Jensen, Sølvi Helseth

**Affiliations:** 1 Faculty of Health Sciences, Oslo and Akershus University College of Applied Sciences, Oslo, Norway; 2 Norwegian Knowledge Centre for the Health Services, Oslo, Norway; 3 Regional Centre for Child and Adolescent Mental Health, Oslo, Norway; 4 Institute of Health and Society, University of Oslo, Oslo, Norway; Universidade de Brasília, Brazil

## Abstract

**Background:**

Using the conceptual framework of shared decision-making and evidence-based practice, a web portal was developed to serve as a generic (non disease-specific) tailored intervention to improve the lay public's health literacy skills.

**Objective:**

To evaluate the effects of the web portal compared to no intervention in a real-life setting.

**Methods:**

A pragmatic randomised controlled parallel trial using simple randomisation of 96 parents who had children aged <4 years. Parents were allocated to receive either access to the portal or no intervention, and assigned three tasks to perform over a three-week period. These included a searching task, a critical appraisal task, and reporting on perceptions about participation. Data were collected from March through June 2011.

**Results:**

Use of the web portal was found to improve attitudes towards searching for health information. This variable was identified as the most important predictor of intention to search in both samples. Participants considered the web portal to have good usability, usefulness, and credibility. The intervention group showed slight increases in the use of evidence-based information, critical appraisal skills, and participation compared to the group receiving no intervention, but these differences were not statistically significant.

**Conclusion:**

Despite the fact that the study was underpowered, we found that the web portal may have a positive effect on attitudes towards searching for health information. Furthermore, participants considered the web portal to be a relevant tool. It is important to continue experimenting with web-based resources in order to increase user participation in health care decision-making.

**Trial Registration:**

ClinicalTrials.gov NCT01266798

## Introduction

The active involvement of healthcare users (hereafter referred to as ‘users’) is argued in respect for individual autonomy, as a critical component of sustainable health and healthcare [1], [2], and as central to evidence-based practice [3]. However, effective participation is dependent on access to research-based information, and skills that would enable users to obtain, understand, evaluate, and act upon the information available [2]. Such health literacy skills include basic reading and numeracy (functional literacy), as well as critical and social skills [2], [4]. Health literacy is described by the World Health Organization as the main desired outcome of health education [5], as an asset in itself, and as a public health issue [2], [5]. Through health literacy, it is argued that people are able to take better control of their own lives and health, including the personal, social and environmental determinants of health [2], [6], [7]. In a systematic review of the evidence, low health literacy levels were associated with poorer health, increased health care utilisation, inappropriate drug use, and the low uptake of disease prevention services (such as vaccinations) [8].

Although access to health information has been improved greatly by new information technologies, evidence-based information is not readily available to the lay public [9], [10], [11]. Studies have found that users may be overwhelmed and frustrated by the vast amount of information available and unsure about who or what they should trust [12], [13]. Moreover, people struggle to understand and critically appraise health information, do not effectively check the accuracy of health information they find and overrate the trustworthiness of such information [13], [14], [15], [16], [17], [18]. Specifically, people are unfamiliar with the principles of medical and health related research and concepts such as randomisation, risk, uncertainty, and causality [17], [19], [20], [21], [22], [23]. Research has also shown that many people are sub-optimally involved in decision making, unaware of their rights or of treatment alternatives, and uncertain about what they need to ask their health care provider [24], [25], [26], [27], [28].

Theoretical and empirical studies suggest that the development of initiatives targeting critical and interactive skills among users is needed, and that these efforts should be evaluated in order to inform practice [4], [29], [30]. Essential skills includes basic reading, writing and numeracy skills (*functional* or *fundamental literacy),* but also critical and social literacy skills, including scientific literacy, civic literacy and cultural literacy [4].

Coulter and Ellins' comprehensive review of the evidence indicates that interactive online interventions may be effective strategies for health education [30], [31], [32]. This method of learning is associated with high levels of user satisfaction [30] and may also be more cost-effective and flexible compared to traditional methods of health education [32]. The content can be also be easily updated and made available to all.

Inspired by these findings we developed a web portal, with the aim of improving the public's access to evidence-based health information and health literacy skills. What health literacy skills really entail has been conceptualised in many different ways. We used the multi-dimensional model formulated by Zarcadoolas and colleagues which contains four central domains: fundamental literacy (reading, writing, speaking and working with numbers), science literacy (understanding and using science and technology), civic literacy (skills and abilities that enables awareness, participation and involvement), and cultural literacy (skills and abilities to recognise, understand and use beliefs, customs, world-views and social identities) [4]. The web portal was designed from a public health perspective to target both healthy people as well as patients, and to be used either independently or in consultation with health providers. The web portal was intended to be used by those who are interested in searching for health information, who would like to know more about medical and health-related information, or need support in decisions related to health. Its content and key intervention targets were informed by extensive literature searches as well as explorative pre-studies with input from people within our target audiences, including focus groups and a questionnaire study based on the Theory of Planned Behaviour (TPB) [24], [33], [34], [35]. Three key barriers to obtaining information were identified; not knowing where to find reliable and relevant information, the inability to understand and critically appraise health information and the inability to exchange information in consultations [35]. The content of the web portal was tailored to address these barriers by facilitating specific domains of health literacy through a choice of evidence based strategies [35]. The web portal was developed within the conceptual framework of the shared-decision making model and evidence based practice, encouraging participation and emphasising the importance of that decisions should be based on the best available evidence [1], [3]. Using illustrations of typical healthcare topics, the web portal focused on *how* research is conducted and *why* this is important rather than just reporting conclusions and expert interpretations. Generic and non-disease specific in focus, the web portal was designed to be applicable to a range of healthcare decisions and settings, and included three facilitators or tool-sets to address each of the main barriers to obtaining information:

1. Access to medical and health-related research databases, an introduction to research methods, the principles of science (based on the steps of the evidence based practice model) and levels of evidence synthesis [36], [37].
2. A checklist for critically assessing health information (DISCERN) [38] and information about why critical assessment is important.
3. A checklist for consultations with health care providers [39] and information about what decision making related to treatment and screening entails.

The development and content of the web portal is described in more detail in another paper [35], or can be viewed online at www.sunnskepsis.no. An overview of the targeted barriers, the content of the intervention, the hypothesised corresponding health literacy domains targeted are presented in table 1.

**Table 1:** Overview of the intervention components, corresponding hypothesised health literacy domains targeted and measurements to evaluate these.

Barriers identified in pre-studies and literature search	Facilitators/content of intervention	Health literacy domains*	Evaluated in pragmatic trial
**All**	Shared decision making (promoting an active role) and evidence based practice as conceptual framework (promoting evidence based decisions)	Civic literacy (system and relationships)	TPB **(attitude and subjective norms associated with search)/ PAM***
		Science literacy	
**Inability to understand and critically appraise health information**	Improving critical appraisal skills	Science literacy Examples: Validity, uncertainty, causality	Searching task/ critical appraisal task/ TPB (perceived behavioural control and attitudes towards search)
	Introduction to scientific concepts and (checklist for) evaluating trustworthiness of health information	Functional literacy (numeracy). Example: Understanding risk	
		Civic literacy (media literacy) Examples: How research and scientific discourse are presented in the media	
**Not knowing where to find reliable and relevant information**	Improved access to reliable research based sources of health information	Science literacy. Examples: Basic study designs and assessment of relevance	Searching task/ TPB (perceived behavioural control and attitudes towards search)
	Introduction to searching for evidence based information (adapted EBP-model)	Civic literacy (media literacy). Examples: Search strategies, publication types and sources	
**Inability to exchange information in consultations**	Enabling exchange of health information	Science literacy	PAM
	Introduction to clinical decision making and checklist for the consultation	Civic literacy (system and relationships)	
		Cultural literacy (understanding of concepts used in decision making about health care)	

* Health literacy domains based on the model by Zarcadoolas and colleagues [4], **Theory of planned behaviour, ***Patient activation measure.

The objective of this study was to evaluate the effects of this web portal intervention compared to no intervention in a real life setting on:

Beliefs about searching for health information and overall activation (participation).
Searching for research-based information and the development of critical appraisal skills.

In addition, we also wanted to get feedback from the participants on their satisfaction with the web portal.

## Methods

The protocol for this trial and supporting CONSORT checklist are available as supporting information; see Checklist S1 and Protocol S1.

### Design

The study was a pragmatic parallel-trial in which one group received access to the portal and its tools (the intervention group) while the other group received no intervention (the control group).

#### Participants and recruitment

Our intention was to include typical users for the web portal in the participant sample. In addition, we wished to increase the probability that the portal would be used by participants in association with visits to health professionals during the trial. Parents with children under the age of 4-years of age were therefore targeted. At this life stage, parents are typically having many questions about treating and preventing health problems. They are also healthcare users with the highest number of health visits per year both for themselves (a mean number of visits per year of 4. 6), and for their children (mean number of visits of 3) [40]. Such parents are also statistically more likely to search for health information online [41].

#### Sample size justification

Sample size calculations should be based on assumptions about expected underlying population event rate and minimum detectable difference in means (and standard deviation of the response) in previous studied populations [42]. Few studies, however, have targeted the publics' health literacy skills including domains other than functional literacy. Thus, for the outcomes included in this trial we had very little previous experience to rely on. Considering that the intervention was passive in nature, we anticipated the effects to be modest. We assumed a conservative minimum detectable difference of means (amounting to a one point difference on seven point scale with a standard deviation of 2,1) on the outcome ‘beliefs about searching’. These assumptions were supported by the means and standard deviations observed in the piloting phase and in the validation study of the TPB questionnaire [34]. Furthermore, we based our sample size calculations on a power of 0.80, a level of significance of 0.95 and the use of a two-sided t-test for statistical analysis. Hence, the required sample size was estimated as 140 persons.

#### Sources and methods of recruitment

Information about the study was distributed at maternity and child health centres, in online advertisements on social media networks, on Internet sites such as Google, and discussion forums for parents. Those who were interested and wished to participate were directed to a recruitment web page.

#### Informed consent and inclusion of participants

**Figure 1:**
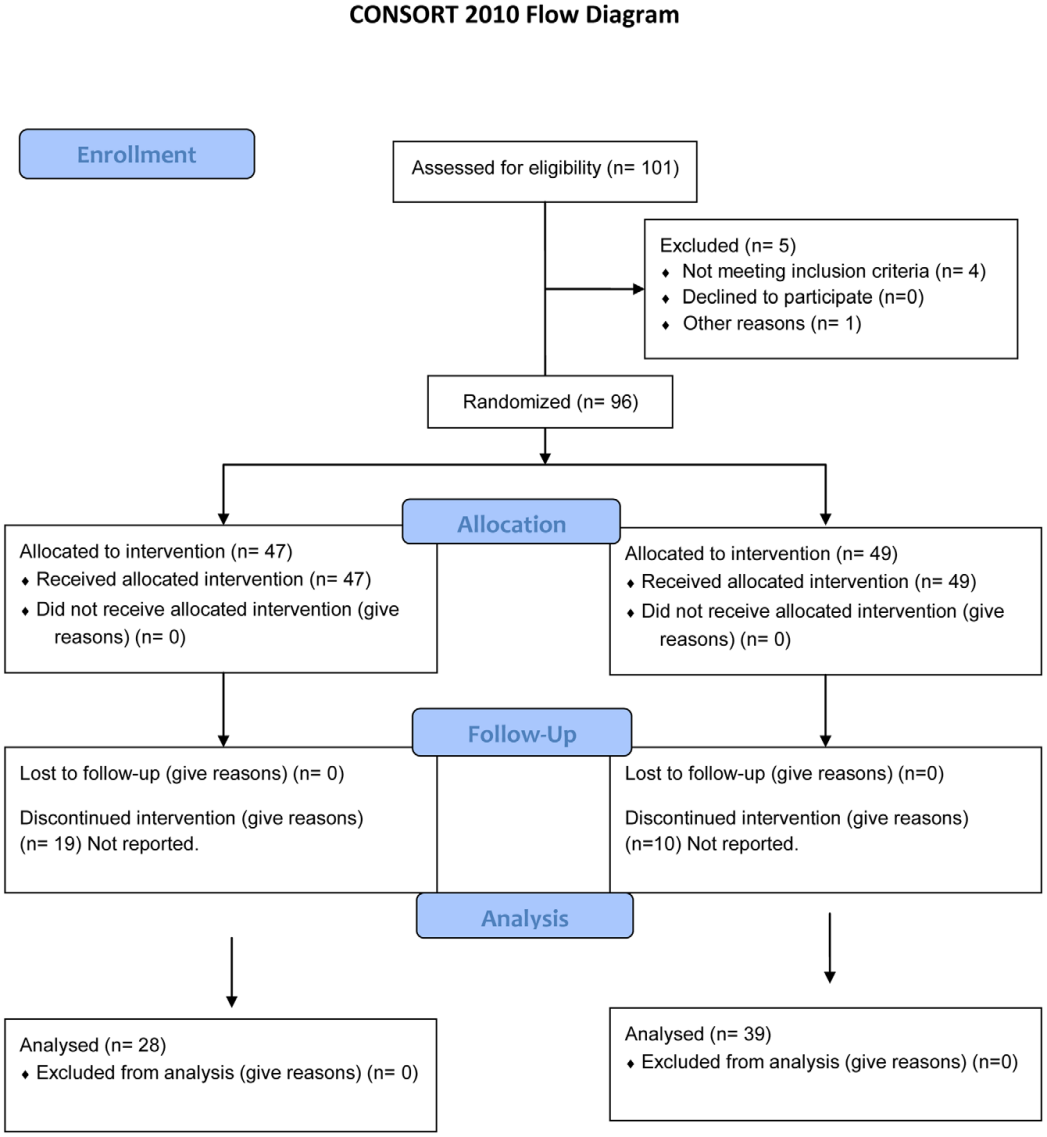
CONSORT 2010 flow diagram.

People who expressed an interest in participating received information about the study, were asked to give written consent to participation, and directed to an online questionnaire for inclusion criteria screening. Participants were excluded if anyone else in their household was already participating in the study (to ensure that participants were blinded and to protect against potential sample contamination) and if they did not have children aged <4-years. If a participant did not meet these inclusion criteria, he or she was sent automated feedback describing the reasons why they were ineligible to participate (see Figure 1 for the CONSORT flow diagram).

#### Randomisation methods and allocation concealment

Those who met the inclusion criteria were randomised using a simple randomisation procedure developed by SPSS. The study was single blinded in that participants were not informed about the study group to which they were allocated. All participants were told that they would be participating in testing a new web portal resource but that they would receive initial access to the portal at different times. All participants were given the same information and treated equally through the use of automated online systems and standardised emails.

#### Intervention delivery

**Figure 2:**
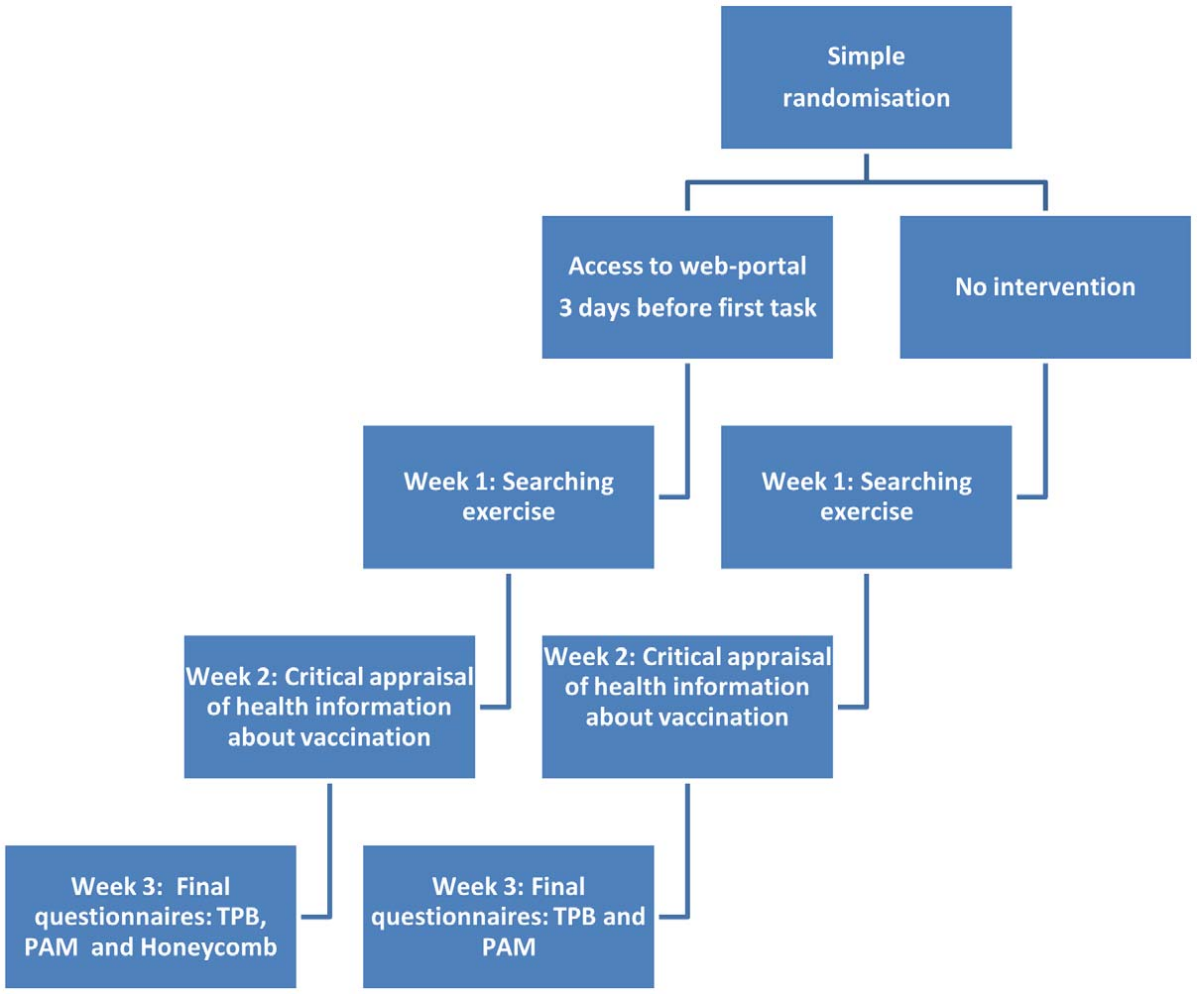
Overview of the study design.

All information was delivered online, and data were collected using an online questionnaire system. Participants were sent tasks by email (Figure 2) at three different times. The intervention group was allowed access to the web portal immediately after randomisation through an email containing the URL, and given three days to explore its content and tools before receiving the first task. Each of the tasks corresponded to the web portal's three main content sections, namely: the improved use of research based information, improved critical appraisal of health information, and improved beliefs about participation (search and activation).

The purpose of the first task, the searching task, was to evaluate the degree to which participants used research based sources to obtain healthcare information. Participants were asked to formulate a question and to answer it by searching for online information. The intervention group was not asked specifically to use the web portal for this task and were thus free to search for information using any resource they felt would be useful, including the web portal.

The purpose of the second task, the critical appraisal task, was to evaluate participant critical appraisal skills related to health information. All participants were asked to rate the trustworthiness of an online article describing how to prevent swine flu, and that included information about vaccinations and alternative therapies. The intervention group was asked explicitly to use the full DISCERN critical appraisal tool which was provided on the web portal [38]. DISCERN is an instrument for patients and other lay-users of health information and is designed to allow them to evaluate the reliability of written information about treatment choices [43]. Swine flu was chosen as the subject for this task for a number of reasons. All vaccinations are voluntary in Norway, and related healthcare decisions were therefore seen as a topic that would be of concern to all parents. Moreover, there has been considerable discussion about vaccinations in the media over the last few years, characterised by strong and often conflicting viewpoints [23], [44], [45], [46], including debate about swine flu vaccinations [47], [48]. We therefore viewed this topic as having considerable potential interest to participants. The specific material we chose for evaluation was taken from a health information site identified using a Google search and was typical of the kind of information available on sites used by lay-people searching for health information.

The third task, reporting of beliefs about search for health information and activation, was designed to explore potential differences between the intervention and control groups in terms of beliefs about health information searches (attitudes, social expectations, perceived behavioural control and intention to search). It also served to explore differences in overall levels of activation. In this final task, the intervention group was also given four additional questions in order to evaluate their satisfaction with the web portal.

Each of the tasks was sent to participants at weekly intervals. This timing and the overall length were decided on for two reasons: firstly, participants were provided with sufficient time to complete the tasks at their own pace. Secondly, longer periods or shorter intervals between tasks could possibly have had potentially negative consequences on the response rate and result in participation fatigue. At the end of the study, after all data had been collected, the control group was also given access to the web portal. The time-frame for data collection was pre-specified, beginning March and lasting throughout June 2011.

#### Participant retention

Basic non-sensitive background information and email addresses were kept on file to allow for a descriptive analysis of losses to follow-up. One automatic reminder per task was sent to participants if they failed to respond within six days.

#### Missing data

No attempt was made to impute missing data due to no valid assumption to base this imputation on. All analyses were performed using available data, with all participants being analysed in the group to which they were randomly assigned.

#### Outcome assessment and analysis

Few tools are available for evaluating improvements in health literacy skills, and most map only general reading and numeracy skills (in other words, only functional literacy) [49]. Our intervention was intended mostly to target and evaluate critical and social skills and we identified no single available instrument suitable to achieving these goals. The outcomes of this study were therefore evaluated using a selection of instruments which, when combined, were considered adequate to evaluating most of the important health literacy skills targeted by our intervention. The main outcomes and corresponding instruments are described in further detail below (see also table 1) and include both actual behaviour as well as behavioural beliefs. All data were automatically exported into SPSS via the online data collection program.

For the searching task the outcome was evaluated by categorising the accessed Internet material (identified by hyperlinks) as information that either had or had not been based on research. The information was considered to be ‘research-based’ if it took the form of a report about original research (e.g. a primary study) or summarised research that had been based on explicit and systematic criteria (e.g. a systematic review or decision support). The information was excluded if no references were provided or identified, and if there were no explicit systematic criteria related to how and why any included references had been chosen. The material (identified by hyperlinks) was categorised by two independent and blinded researchers with training in the field of evidence-based practice and evidence synthesis. Their conclusions were then discussed further with the lead researcher. It was expected that research based information would be found by more participants in the intervention group than the control group. This hypothesis was tested by calculating the relative risk and corresponding confidence intervals.

For the critical appraisal task we used DISCERN appraisal tool (item number 16) to compare overall respondent ratings of the swine flu information [43]. This tool measures the respondent's overall rating on a scale of 1–5, where a score of 1–2 indicates ‘low quality’ (serious or extensive shortcomings), 3 indicates ‘moderate’ quality (potentially important but not serious shortcomings) and a rating of 4–5 indicates that respondents felt the material to be of ‘high quality’ with only minimal shortcomings [43]. The study group's mean value information rating was measured against ratings of the same material made by two blinded external experts with professional research and healthcare backgrounds. Based on results from previous studies using DISCERN, the mean overall score of the intervention group was expected to be closer in value to the experts' rating than the control group [38]. We also expected that the overall quality rating made by the intervention group would be lower, given that evaluations based on explicit criteria tend to be more critical compared to personal opinion [50]. The effects of the intervention were measured by calculating the mean differences between the groups and applying one-sided t-tests.

For measuring beliefs about intention to search for health information and activation we used two instruments. A questionnaire was designed specifically for the purpose of evaluating beliefs about searching for health information, based on the TPB [34]. The Patient Activation Measure (PAM) was used, with permission from Insignia Health, to evaluate overall activation and participants' self-management abilities [51].

**Figure 3:**
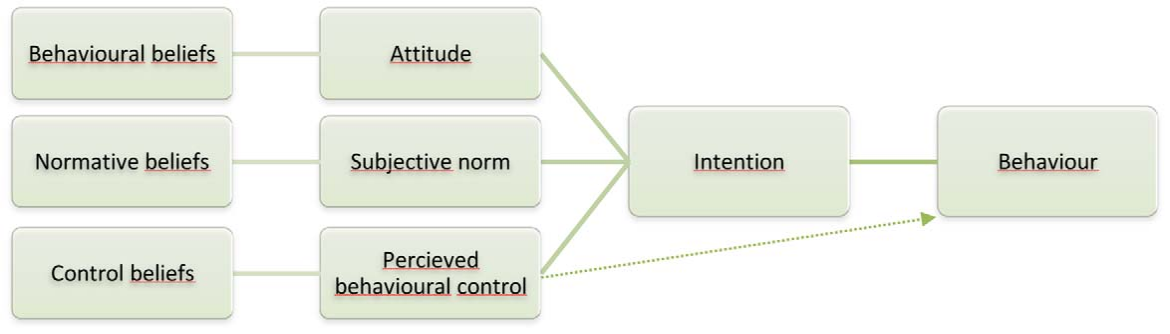
Model of the theory of planned behaviour by Ajzen 1991.

The TPB is a social cognition model rigorously tested and widely used to predict behavioural intentions [52], [53], [54]. According to this model, there are three cognitive variables that can predict behavioural intentions (Figure 3) [52], [55]. These are:

1. Attitudes towards the behaviour (beliefs about the consequences of the behaviour, and positive or negative judgements about these consequences).
2. Subjective norms about the behaviour (a person's own perception of social pressure or expectations and motivation to comply).
3. Perceived behavioural control (how much a person has control over the behavior and how confident a person feels about being able to perform or not perform the behavior).

These three variables are measured using direct and indirect measures [52]. While the direct measures describe the overall beliefs associated with behavioural intentions, in this study of intention to search for health information, the indirect measures represented the specific beliefs that underlay the overall assessments and were seen as explanatory factors. To evaluate the influence of the intervention on the direct and indirect TPB components, we calculated the differences in mean overall scores and specific beliefs using a two-sided t-test. The minimum and maximum mean composite score for direct measures were 1 to 7, −21 to +21 for specific indirect measures and −63 to 63 for overall composite indirect score for subjective norm and perceived behavioural control, and −84 to +84 for attitude (where higher values indicate more favourable attitudes, greater social pressure and higher perceived behavioural control). The intervention group was expected to have stronger beliefs than the control group.

To explore the effects of the intervention on predicting intention to search, the dependency between ‘intention’ and the composite direct measures from the TPB questionnaire was investigated using a multiple regression model in which ‘intention’ was the dependent variable. Group assignment (web portal vs. no intervention), and the three composite scores from the TPB model (namely ‘Attitudes towards the behaviour’, ‘Subjective norms’ and ‘Perceived behavioural control’) were entered as independent variables. In addition, we estimated the effect of the intervention on the dependency between the three composite scores and the intention by entering the interaction terms between group assignment and each of the three composite scores as independent variables in the model. Furthermore, the TPB model, and consequently the questionnaire, consists of several operationalisations of theoretical constructs with certain assumptions about interrelationships between these (Figure 3). These relationships were explored by computing simple bivariate correlations using Pearson's r. The direct TPB measures are hypothesised to be positively correlated with intention, but the direct measures may also be interrelated as these are not seen as categories independent of each other [55]. The questionnaire is available upon request by contacting the authors.

Overall activation was measured using the Patient Activation Measure (PAM), a validated instrument applicable to both patients and healthy people [51], [56]. This instrument includes four key domains (believing the patient role is important, having confidence and knowledge necessary to taking action, actually taking action to improve one's health, and staying the course under stress) which are measured by a total of 13 items [56], [57]. Overall activation is scored on a rating from 0–100, where 100 indicates high activation and 0 indicates no activation [56], [57]. We anticipated that activation levels in the intervention group would be higher than activation levels for the control group. To evaluate this outcome, a two-sided t-test was used to measure and test the difference in mean overall score.

For measuring satisfaction with the web portal, we obtained user feedback from the intervention group about the web portal. Our evaluation of this was based on the Honeycomb model [58], a useful instrument applied to measurements of Internet site user experiences [59]. The model encompasses seven domains to assess whether a website is accessible, usable, credible, valuable, findable, desirable and useful [58]. In our study, questions were used to assess three of these measures of user satisfaction (credibility, usefulness and usability), each measured on a satisfaction scale from 1–7 (higher values indicating greater satisfaction). The following data were then summarised for each domain: mean values, standard deviation, median, and interquartile range.

### Safety monitoring and adverse events

A tool to encourage participation could create potentially unnecessary pressure on users. This domain was captured using the TPB questionnaire (subjective norm). Other adverse effects were deemed unlikely.

### Ethical aspects

Data were treated anonymously, and ethical approval was granted by the Norwegian Social Science Data Services (NSD), and Regional Committees for Medical and Health Research Ethics (REK). The trial was registered under the ClinicalTrials.gov identification number NCT01266798.

## Results

### Description of study participants

Four participants were excluded from the study because they did not meet the inclusion criterion of having children aged <4-years. One respondent provided incomplete contact details. In total, 96 participants were included, of which 47 were randomised to the intervention group and 49 to the control group (Figure 1). The overall response rates for the intervention group and control group were 60% (n = 28) and 80% (n = 39) respectively (Table 2). Those who chose to complete the first task generally continued throughout the whole study. The number of parents per outcome was 28 in the intervention group and 39 in the control group for the searching task (use of research), 27 in the intervention group and 39 in the control group for the critical appraisal task and 28 the intervention group and 39 in the control group for the beliefs about search and activation outcome.

**Table 2:** Description of participant characteristics.

	Intervention	Control
Response rates total	60% (n = 28)	80% (n = 39)
% Men	20%	22%
% Females	80%	78%
% Primary school	9%	0%
% High school	16%	12%
% 1–3 years of college/ University education	22%	22%
% 3+ years of college/ University education	53%	66%

### Use of research

Two research-based sources were identified in the intervention group, and one in the control group. The relative risk was 2.8 (CI 0.3–29.2) (p = 0.39).

### Critical appraisal

The mean rating of the information was 2.41 (SD 0.80) by the intervention group and 2.44 (SD 1.02) by the control. The mean difference was –0.03 (p = 0.904).

The difference between the expert rating (rated as 1) and the rating of the intervention group was 1.40, and for the control group was 1.43 (difference = −0.03; p = 0.904).

**Figure 4:**
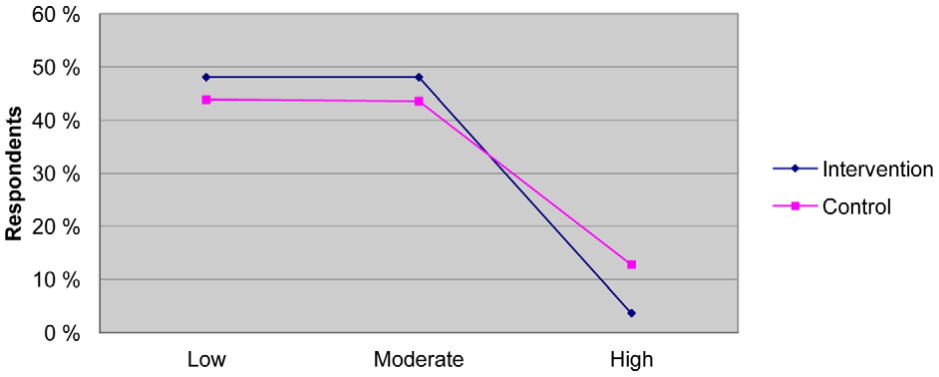
Rating of quality across study groups.

The distribution of the ratings across the two groups was not significantly different (Figure 4; Pearson Chi-Square = 1.605, p = 0.448).

### Beliefs about searches for health information and activation

A statistically significant difference of 0.63 was found for overall attitude towards search: 0.6 (p = 0.03) in favour of the intervention group (see Table 3 for mean scores). The mean differences for the overall subjective norm (−0.2; p = 0.49) and perceived behavioural control (0.41; p = 0.15) as well as specific beliefs (all three p-values >0.25) related to search, were not statistically significant.

**Table 3:** Distribution of means and differences between groups.

Variable	Mean Intervention (SD)	Mean Control (SD)	Mean difference (95 % CI)	P-value
Intention*	6.1 (1.1)	5.8 (1.1)	0.3 (−0.2 to 0.9)	0.20
Direct attitude*	5.8 (1.1)	5.2 (1.2)	0.6 (0.1 to1.2)	0.03
Direct subjective norm*	3.4 (1.3)	3.6 (1.2)	−0.2 (−0.8 to 0.4)	0.49
Direct perceived behavioural control*	5.6 (1.1)	5.3 (1.1)	0.4 (−0.2 to 1.0)	0.15
Overall indirect attitude**	53.7 (24.2)	50.8 (24.8)	2.9 (−9.3 to 15)	0.64
#1 Provides insight***	14.3 (6.7)	14.0 (6.5)	0.3 (−2.9 to 3.6)	0.83
#2 Useful as background in consultations***	12.8 (7.8)	11.4 (7.7)	1.4 (−2.5 to 5.3)	0.48
#3 Helpful if unsure in health decision***	12.4 (6.8)	12.4 (7.0)	0 (−3.4 to 3.4)	0.99
#4 Provides additional information If incomplete information from health***	14.1 (6.8)	13.0 (7.4)	1.1 (−2.4 to 4.7)	0.52
Overall indirect subjective norm**	13.9 (14.5)	10.3 (12.5)	3.6 (−3.0 to 10.3)	0.28
#1 Family and friends***	8.1 (6.5)	6.1 (4.9)	2.0 (−0.8 to 4.8)	0.15
# 2 Health professionals***	0.7 (6.9)	1.0 (7.4)	−0.3 (−3.9 to 3.3)	0.89
#3 Other social groups (colleagues, patient organisations)***	5.1 (5.1)	3.2 (3.5)	1.8 (−0.3 to 4.0)	0.09
Overall indirect perceived behavioural control**	−4.4 (13.4)	−3.1 (17.3)	−1.3 (−9.1 to 6.6)	0.74
#1 Difficult to attain an overview***	−0.3 (6.5)	−0.3 (7.2)	0 (−3.4 to 3.5)	0.99
#2 Not possessing knowledge***	−0.8 (5.4)	−1.2 (6.6)	0.4 (−2.6 to 3.4)	0.79
#3 Time consuming***	−3.3 (5.9)	−1.6 (7.0)	−1.7 (−5.0 to 1.5)	0.30

* Mean minimum and maximum score possible is 1 to 7 (stronger beliefs indicated by higher) **Mean minimum and maximum score possible is −63 to 63 for subjective norm and perceived behavioural control, and −84 to +84 for attitude. ***Mean minimum and maximum score possible is −21 to 21.

The dependency between the direct composite measures from the TPB and intention to search was found not to differ significantly across the groups (p>0.1 for all three composite scores). The TPB constructs' overall prediction of intention to search across the complete sample was approximately 37% of the observed variation in the intention to search. Attitude was the most important positive predictor of intention (b = 0.51; p<0.002), whereas the predictive strength of subjective norm and perceived behaviour control was −0.15 (p = 0.25) and −0.06 (p = 0.72) respectively.

Direct attitude and perceived behavioral control had a statistical significant and positive correlation with intention (Pearson's r = 0.56 and 0.41 respectively), and were also found to be inter-correlated (Pearson's r = 0.59) (all p<0.001). Subjective norm had a non-statistically significant negative correlation with intention (Pearson's r = −0.1; p = 0.92), and non-statistically significant positive correlation with attitude (Pearson's r = 0.18; p = 0.15) and perceived behavioral control (Pearson's r = 0.69; p = 0.58).

The overall activation (PAM) score was 66.5 in the intervention group and 61.9 in the control group, and the mean difference was 4.61 (p = 0.20).

### Satisfaction with the web portal

The mean usefulness rating of the web portal was 4.71 (SD 1.11), mean usability 4.14 (SD 0.97) and mean credibility 4.75 (SD 0.93) (see table 4 for respondents' rating).

**Table 4:** Satisfaction with the web portal.

N = 28			Usefullness	Usability	Credibility
Mean			4.71	4.14	4.75
Median			5.00	4.00	5.00
Standard deviation			1.117	970	.928
Percentiles	25		4.00	3.00	4.00
	50		5.00	4.00	5.00
	75		6.00	5.00	5.00

* Mean minimum and maximum score possible is 1 to7 (stronger satisfaction indicated by 7).

## Discussion

### Study limitations

This study had limitations but also appreciable strengths. Participant recruitment can be a significant obstacle in the research process, and this proved to be a challenge in our study. Given the resources available, we were unable to recruit the number of participants suggested by the power-analysis. The response rates were also lower than expected. Considering that the content of the web portal and tasks included in the trial may be considered as challenging or difficult by many parents, particularly by those with lower health literacy, a higher drop-out could have been expected among those with lower education. Although the correlation between health literacy and education is not perfect, it is a relevant predictor [8]. Background data information about those who dropped out of the study revealed no statistically significant differences in sex or education levels, compared to those who did not drop out which is a positive finding. There was, however, a small but borderline statistically significant (p<0.06) difference between the intervention and control group in terms of loss to follow-up: more were seen to drop out of the intervention group (Table 1). The difference in drop-out between groups may indicate that people joined the study primarily in order to receive access to the portal. Once they had access, they may have dropped out before the first task was given. Of those who did participate in one or more of tasks, the background characteristics across the intervention group and the control groups showed that participant distributions by sex and education were very similar. The mean level of education, however, was slightly higher in the control group. The fact that the loss to follow-up mainly happened between the time of the first screening questionnaire and the time of the first task may indicate that the tasks included in the trial were considered too extensive.

Using an online questionnaire system was a time-saving and cost-efficient data collection method. It allowed for the confidential treatment of data and provided an opportunity to use automatic systems for keeping track of response rates, sending out reminders, sending notifications directly to participants if data were missing, and to export the data directly into Excel and SPSS. In this way we reduced the risk of potential errors inherent in manual data entry, ensured that all communications were standardised, and that both groups were treated equally.

### Intervention effects on critical skills

The effects of the intervention on the critical skills of participants were evaluated by means of a searching task and a critical appraisal task. Only minor improvements in the intervention group were identified by both tasks and these differences were not statistically significant.

The number of research-based sources reported by participants in the searching task was very low in both study groups. Interestingly, the majority of participants in the intervention group chose to use sources not included in the portal. This may imply that the respondents preferred to rely on sources with which they were already familiar, or that that the three-day interval between being given access and having to perform the first task, may have been too short to allow them to become familiarised with the portal itself.

In the analysis, the sources were categorised based on pragmatic yet strict judgments regarding whether the information was research-based or not. This proved to be difficult, particularly because few of the sites provided references or indicated what their information was based on. This applied even to information provided by government and public health organisations. Consequently, information that was research-based may have been excluded if it lacked appropriate references. One source, for example, which was originally excluded because it lacked references, was then re-included in our study based on the knowledge of our lead researcher (this material was the Norwegian translation of BMJ's Best Practice Patient information). That online health information is often unclear, incomplete or even misleading is supported by reviews of online literature [9], [10], [45] and underlines the importance of providing users with easily accessible research-based information and the need to improve critical appraisal skills. But it also indicates that those who present such information on the Internet should critically review their own publishing practices and place greater emphasis on transparency and sources of their information.

### Beliefs about participation and intervention effects

Beliefs about participation were evaluated using a TPB questionnaire that addressed intention to search and underlying factors, and the PAM questionnaire describing overall activation. The overall score for beliefs about participation (based on both the TPB and PAM) was high in both groups, indicating that participants viewed taking a more active role favourably. When comparing the two groups, we found small statistically non-significant improvements in favour of the intervention group with both the instruments indicating higher intention to search and higher activation.

In the TPB questionnaire, both groups were also found to have strong favourable attitudes towards searching for health information. Whereas both groups reported high overall perceived behavioural control, their responses to specific beliefs highlighted the fact that certain aspects of searching were perceived as being slightly difficult. The overall social pressure to search was moderate. Of particular interest was the fact that parents experienced very little pressure or expectation from health professionals who might have been anticipated to have a central role in this. Our findings are similar to those from other studies showing that independent searches for health information are rarely discussed, facilitated or addressed during healthcare consultations [60], [61], [62], [63].

The mean differences of direct measures across the groups showed a statistically significant difference in favour of the intervention group in terms of overall attitude 0.6 (p = 0.03). This suggests that the web portal may be a useful tool for improving attitudes towards searching for health information, and is an interesting finding in view of the fact that attitude was also found to be the most important predictor of intention to search in both groups. That we found favourable attitude to be an important factor associated with searching for health information is supported by other studies [13], [60], [64], including another study in which the same questionnaire was used [34]. Perceived behavioural control was only a weak and not statistically significant predictor of intention to search but was found to have a strong correlation with both intention and attitude. Thus, although the user feeling of being in control of searching does not directly predict intention, it may still be an important underlying variable. Subjective norm was also found to have a weak relationship with intention and this finding supports earlier research showing that social expectations or pressure do not appear to be an important or positive predictor of intention to search [34].

We identified no statistically significant difference between the groups in terms of the effect of the intervention on the prediction of intention to search. The TPB components' overall prediction of intention to search was satisfactory and generally consistent with other studies in which the TPB had been used to predict behavioural intentions across a range of health topics [53].

### Satisfaction with the portal

The satisfaction with the web portal was good. This is encouraging given that the purpose of the web portal, and the concepts introduced, were potentially novel to most respondents. A challenge in this project was that although we wanted to improve user access to evidence-based information we also wanted to improve knowledge and skills. When developing the web portal, a compromise was therefore reached between usability (how easy it is to access reliable health information) and educational intention (reflected for example by how people are routed through the web pages). Consequently, the web portal may potentially demand more of its users relative to traditional sources of health information. Despite this, the results of this study indicated that we may have come a long way towards achieving this balance correctly.

In the next phase of our research, the web portal will be made publicly available and will be search optimised. This will mean that when people search for health information using a general search engine, the web portal will range high on the list of hits and, in this way, will contribute to making evidence-based health information more available to the general public. The web portal is also in continuous development, and more features will be considered for inclusion. Ideas for expansion include a discussion forum about science and research, and a blog addressing current issues related to medical and health related information in the public debate.

### Conclusion

This study was a pragmatic trial conducted in a real life setting. The recruitment of participants was challenging, the response rates were somewhat low and the intervention effects smaller than expected. Although this resulted in the study being underpowered, we found improvements on attitudes towards searching for health information, a variable identified as the most important predictor of intention to search. The relevance of the web portal to users was confirmed by the fact that the participants considered the web portal to have good usability, usefulness and credibility.

### Implications for practice

It is vital that health practice and decision making should be based on the best available, current, valid and relevant evidence. Recognising that users should play a central role in evidence-based practice, people should be encouraged to take on an active role. Moreover, resources such as web portals should be made available in order to facilitate greater access to, and critical use of, research-based information.

Future efforts should aim at experimenting more with web-based resources in order to encourage user involvement in health care. Large samples are needed to identify more robust results. Furthermore, online resources alone may not be sufficient to improve health literacy skills effectively. More intensive interventions could include the use of the web portal during consultations with health providers, or as part of evidence-based practice courses for users such as patient representatives who have a particular interest in healthcare issues.

## Supporting Information

Checklist S1 **CONSORT checklist.**
(DOC)

Protocol S1 **Trial protocol.**
(DOC)
