# Supplementary material for: Evaluation of a Web Portal for Improving Public Access to Evidence-Based Health Information and Health Literacy Skills: A Pragmatic Trial
Source: PLoS One. 2012 May 31;7(5):e37715. doi: 10.1371/journal.pone.0037715 (PMC3365121; doi:10.1371/journal.pone.0037715)
Supplement: Protocol S1 — Trial protocol. (DOC) [file pone.0037715.s002.doc]

**Astrid Austvoll-Dahlgren, Sølvi Helseth, Arild Bjørndal, Jan Odegaard-Jensen**

**Protocol**

Effects of a patient directed web-portal to improve health literacy skills: a randomised controlled trial

**Summary**

Access to the best available evidence, and the ability to obtain and understand such information is seen as necessary to protect the public’s interests and critical to empowerment, but is also a precondition for participation in the decision making.

A web portal serving as a generic (non disease- specific) tailored tool was developed in the conceptual framework of shared decision making and evidence based practice to improve the lay- public’s critical and social literacy skills and activation. A randomized controlled parallel trial using a simple randomization procedure will be conducted, including 200 parents of children <4 with internet access. Parents will be allocated to receive either the portal or no intervention. Primary and secondary outcomes include: the ability to find research based information, critical appraisal skills, perceived behavioural control, attitudes and perceived pressure associated with searching for information, and participation.

**Introduction**

Access to the best available evidence, and the ability to obtain and understand such information is seen as necessary to protect the public’s interests and critical to empowerment, but is also a precondition for participation in the decision making . The importance of health literacy has been argued and is often defined as “the degree to which individuals have the capacity to obtain, process, and understand basic health information and services needed to make appropriate health decisions”. In a review of the evidence, health literacy was found to be a stronger predictor of health status than age, income, employment status, education level and ethnicity . Health literacy and participation in health decisions has been associated with improved health outcomes and cost effectiveness but is also advocated on ethical grounds by facilitating equal access to healthcare respecting user autonomy .

Based on pre-studies and a literature search we identified three main barriers to obtain, process, and understand health information; the ability to interpret health information, knowing where to find reliable and relevant information and being able to obtain information in consultations. These barriers will be sought addressed by improving patients’ critical appraisal skills, improving access to reliable sources of health information and providing a checklist for exchange of health information to be used in consultations with health professionals. This will be done using a web portal serving as a generic (non disease- specific) tailored tool developed in the conceptual framework of shared decision making and evidence based practice.

Consumer health informatics is a branch of patient directed interventions that aims to reach patients directly trough new information and communications technologies in an interactive way . In an review of the available evidence and based on a number of reviews and controlled trials, Coulter and colleague found that interventions using new information technologies may improve knowledge, demonstrate high user satisfaction and positive effects on self-efficacy and health behaviour . Evidence also point to that web based patient directed interventions may have benefits over that of conventional means of relaying information

When evaluating the intervention we wanted to focus on a specific health issue in order to contextualize the portal. The case that has been chosen for this trial is childhood vaccinations. The reasons for this are several. Few, if none, are so much in contact with health providers and have questions about how to treat and prevent health problems as parents of small children (49). Childhood vaccinations are voluntary in Norway, and thus this is a health decision that concerns all parents. Moreover, childhood vaccinations is an interesting case considering it has been a hot topic of discussion in the media during the last few years, and many strong and often conflicting opinions have been expressed in the public debate suggesting both important health effects as well as severe side effects .

**Overview of the trial**

*Design*

The trial will done using a parallel design where each group of participants is exposed to only one study intervention.  One group will receive access to the portal and its tools, the other group (control group) will receive no treatment.

**The research question**

What are the effects of patient directed web-portal to improve health literacy skills.

| **P** | Parents of children age <4 |
| --- | --- |
| **I** | Portal |
| **C** | No treatment/ practice as usual |
| **O** | The ability to find research based information, critical appraisal skills, perceived behavioural control, attitudes and perceived pressure associated with searching for information, and participation. |
| **T** | Primary objective:  -Is the portal more likely to improve actual and perceived self- efficacy associated with searching for health information and critical appraisal skills compared to no treatment/ practice as usual?  Secondary objectives:  -Is the portal more likely to improve participation (searching behaviour and activation) compared to no treatment/ practice as usual?  -Is the portal more likely to increase the perceived pressure to search for health information? |

**Trial population**

*Trial site(s) and population(s)*

Parents of children age <4 will be recruited with access to internet.

Considering that the portal primarily is targeted at people who already are searching for information on their own, the intervention will only be relevant for those who already have access to internet. Search for health information has been found to be negatively correlated with age and positively with education . In the Norwegian population<16 years, 2/3 have attained a tertiary education and nearly all households with children have internet at home .

Allowing for that the portal can be used in relations with a health visit, we will limit our sample to include parents of children <4. At this age children are still following the vaccination program but are also on average in contact with a health provider 3 times per year, decreasing with age up to early adolescence where the trend changes back again (49). At the time where parents have children <4, they are also in a peak when it comes to health visits themselves, with a mean number of health visits 4,6 contacts each year (49).

*Exclusion criteria*

Participants will be excluded if one other in the household already participates in the study (due to spill-over effect) and if they do not have children <4. Since the recruitment will be done online, there is no need to screen for access to internet.

*Sources or methods of recruitment*

We wish to recruit participants where they search for information. Thus, participants will be recruited at the maternity and child health centers and through social media and internet sites.

A pamphlet and an ad will be developed based on the participant information material that has been approved by the Norwegian Social Science Data Services (NSD) and Regional Committees for Medical and Health Research Ethics (REK). The pamphlet will be distributed through the maternity and child health centers, whereas online communities and internet sites will be contacted and asked for permission to publish the ad.

The inclusion/ exclusion for study participation will be done online where possible participants will be asked to answer a short online screening form. They will also receive information about the study and asked to consent to participation.

If the participant does not meet the inclusion criterion, he or she will be given an automated feedback describing that they are not eligible to participate in the study.

Those that meet the inclusion criteria will be randomized to receive the intervention or no intervention.

Recruiting

**Action:**

Screening for inclusion using Questback + informed consent

If not meeting inclusion criteria- exclusion

**Action:**

Email explaining reason for exclusion

If meeting inclusion criteria-

Inclusion in study

**Action:**

Participants randomised and sequentially assigned to study groups

*Figure 1- recruiting procedure and allocation procedure*

**Allocation of interventions**

*Methods for randomisation and stratification*

Simple randomization procedure using SPSS will be used.

Recruitment rate will be observable through Questback.

The participants will be sequential allocated.

*Methods for concealment of allocation*

Allocation concealment has been described *as “the process that prevents any trial participant or investigator from knowing in advance the treatment to which subjects will be assigned”* . In order to prevent selection bias participants who meet the inclusion criteria will be allocated based on an automated system. The participants will not be informed about which study group they are allocated to.

**Simple**

**randomisation**

**Portal**

**Week 1:**

Searching exercise

**No intervention**

**Week 1:**

Searching exercise

**Week 2:**

Critical appraisal of health information (Questback)

**Week 2**:

Critical appraisal of health information (Questback)

**Week 3:**

Final questionnaires

(Questback)

**Week 3:**

Final questionnaires

(Questback)

*Figure 2- study design*

**The interventions**

*Description of intervention*

The intervention target for this evaluation was developed as a tailored intervention where pre- studies, a literature search and process evaluation studies helped tailor the content and design as it is today. The portal was tailored to improve the lay public’s’ “healthy skepticism”, or in other words, critical and social literacy skills to assist patients in the decision-making process and for improving access to health information and participation. Illustrated by typical examples which can be found in the news and through patient stories, the portal provides an introduction to research methods, the underlying principles of science and critical assessment tools appropriate for a lay public. Framing and how to disseminate health information is complex. By using scenarios, real-life examples and plain-language terms , the portal focuses on *how* research is conducted, and *why* this is important, rather than just reporting conclusions and expert interpretations. In essence, the portal includes three sections.
1. A checklist for critical assessment of health information
2. Access to research databases for health information and an introduction to research methods and principles of science

3. A checklist to the consultation

The portal was not designed as an online course since this was considered to be a tedious and unlikely approach to take by many users. Instead, an important principle has been that the division of topics should be logically structured for those who want to navigate through menus, but users should also be able to “play” their way through the content by clicking on the titles that look interesting sparking interest. The portal was intended to target the public in general and those who are interested in health information, but also as a suitable tool to be used in the consultation. Generic and applicable to all health decisions, the portal and its included tools will be freely available of no cost, to be accessed online, downloaded or printed out in a user friendly format.

*Intervention delivery*

With the exemption of the first welcoming letter, the intervention will be delivered online, and all responses will be recorded using Questback. At three different stages the participants will be sent an email containing a task.

Each task corresponds with the three main categories of content that the intervention (portal) aims to target; improved search, improved critical appraisal skills and improved activation.

A week interlude has been allowed between each task. This choice of timing was based on two pragmatic considerations; allowing for that the participants may use one week to complete the task at their own pace and time, and at the same time considering the potential consequences on the response rate and participation fatigue that a longer follow-up may have.

*Contact*

With the exemption of the first initial information letter, all communication will take place using email and Questback.

**Outcome assessment**

The intervention is developed within the conceptual framework of shared decision making and evidence based practice where the main goal is to enable patients to reach an autonomous decision they can have confidence in. Thus the main outcome of and intervention within this field is not to measure adherence, rather the focus is on improving efficacy, knowledge and participation .

Health literacy is multi-dimensional and consists of a collection of several sub-literacies; including scientific literacy, media literacy and civic literacy . The portal has been tailored to address some of these skills, including assessing the quality of information, basic knowledge about research and how to search for such information, and a checklist to improve participation and information obtained during consultations. There is no single instrument available to measure changes in all of these domains, thus three instruments will be used that collectively encompass the portals main objectives. The main outcomes and suited instruments are described below.

*Primary outcomes:*

1. The ability to find reliable answers to a health question (measured in week 1)

In week 1 we will ask the participants to formulate a question and to find information that can answer this question. The participants will be asked to copy and paste the internet link to the information they found.

2. Critical appraisal of health information (measured in week 2)

In week 2 we will ask the participants to rate a piece of health information about childhood vaccination. This will done using DISCERNs item number 16, which is a measure where the respondent is asked to rate the overall quality of a piece of health information about treatment (see attachment for the full DISCERN instrument).

DISCERN is an instrument designed for patients and other lay-users of health information to judge the quality of written information about treatment choices . Discern has been developed by experts and lay-people based on the best available evidence and has been thoroughly evaluated . The tool is also translated to Norwegian by NIFAB and has been reproduced in the portal with their permission.

3. Self efficacy and attitudes towards searching for information, attitudes and perceived pressure and participation (week 3)

In week 3 we will make a final assessment of the participants’ efficacy, attitudes and perceived pressure to search for information but also level of activation.

The relationship between attitudes and behaviour is complex, and there are many theories on field . The Theory of planned behaviour approach (TPB) is a method for developing questionnaires that has been rigorously tested and have been proved useful in understanding the attitude-behaviour relationship in health research and when tailoring interventions. The approach proposes a model where intentions are precursors of behaviour, and although there is no perfect relationship between intended behaviour and actual behaviour, intentions has been found to be used as a proxy for behaviour. This discovery was one of the most important contributions of the TPB, in comparison to other available tools on the field.

Based on the theory of planned behavioral manual a questionnaire has been developed and validated in order to inform the development of this portal, but is also well suited as a measurement instrument. Earlier studies have covering a range of topics including speeding prevention , cardiovascular risk prevention and oral health care promotion . The instrument that was developed previous to this study measures perceived behavioral control, attitudes and perceived pressure associated with searching for health information.

The patient activation measure (PAM) has been developed to measure activation . The instrument includes 4 domains; believing the patient role is important, having confidence and knowledge necessary to taking action, actually taking action to improve one’s health, and staying the course under stress .

The PAM has been thoroughly validated, is probably the single most relevant instrument to measure activation following the principles of evidence based practice, shared decision making and health literacy, and is applicable to both patients and other target groups. The instrument is also translated and validated in Norwegian and can be used for both chronic and healthy populations in a prevention perspective .

*Other:*

We will also ask participants to comment on satisfaction with the portal after the other data collection has been submitted. This will be done based on the Honeycomb criteria which were also used in the qualitative evaluation. The honeycomb model is a well-renowned and much used instrument to measure user experience . The Honeycomb-model encompasses seven domains: accessible, usable, credible, valuable, findable, desirable and useful .

**Post-recruitment retention strategies**

*Participant retention*

- Basic non-sensitive background information and email-addresses will be kept on file.
- Two reminders are planned per activity if participants fail to respond.

**Safety monitoring and adverse events**

Choice is crucial for autonomy . But too much choice may leave people feeling overwhelmed and the task may be experienced as de-motivating or simply too difficult resulting decisional conflict . A criticism towards developing a tool such as healthy scepticism may be that it can induce pressure on the public and add to the burden to the enormous landscape of health information already available. Thus a relevant outcome to investigate in this setting is whether the intervention may increase the perceived pressure to search for information. This domain is captured by the theory of planned behaviour questionnaire.

**Data collection and management**

*Data collection and timing*

Participants will be identified through email-addresses and will be asked to fill in a Questback form in three phases:

Week 1- Information letter by post + email with searching exercise and link to online questionnaire

Week 2- Email with critical appraisal exercise containing a piece of childhood vaccination information to be assessed and link to online questionnaire (DISCERN)

Week 3- Email with link to final questionnaires on efficacy, attitudes and perceived pressure associated with search of health information (TPB) and participation (PAM) + satisfaction form for the intervention group (Honeycomb)

Questback easily allows for confidential treatment of data, but is also available at minimal cost.

Data collection is planned ended by July 2011.

*Blinding*

Participants will all receive the same information and will not be informed about which study group they belong to. All participants will also receive the portal; however, the control group will only receive the portal after the final data collection.

*Data entry*

Questback not only allows for registering data but also produces a SPSS data sheet that can be exported directly into SPSS for further analysis.

*Quality control*

A statistician and an advisory group of researchers representing different disciplines and fields of expertise including evidence based practice, knowledge transfer and presentation of research, general medicine, nursing and public health will review the protocol as well as the analysis and final draft of the paper.

**Sample size**

*Sample size justification*

The intervention is considered to be passive and the effect sizes are expected to be conservative. A pragmatic power-analysis was conducted based on the primary outcome measure, considering a minimum detectable difference in means of 1,0 (standard deviation of 2.1) and based on continuous outcome measure results in 70 participants in each group (total n=140). It is recommended that the sample size is inflated to allow for any loss to follow up; leaving the sample size for this trial to be effectively around 200.

*Missing data*

No efforts will be made to impute missing data. All analyses will be performed on available cases, but based on the intention to treat principle (all participants analysed in the group they were randomised to).

**Analysis strategies**

All data will be entered into SPSS and analysed by Astrid Austvoll-Dahlgren under the advice of a statistician Jan Odgaard-Jensen.

- Searching ability

The outcome will be evaluated pragmatically by categorizing the information as based on: 1. research or 2. expert advice/ other. The information will be categorized blindly by two independent researchers. The direction of effect is expected to be positive, in that more participants will find research based information than those in the control group. The hypothesis will be tested using Relative Risks with corresponding confidence intervals.

- Critical appraisal skills (DISCERN)

The mean value rating of the information will be measured against a “standard” rating made by experts on the same information. Two hypothesis regarding critical appraisal skills will be tested:

1. The direction of effect is predicted to be positive, in that the mean overall score of the intervention group will be closer to the standard than the control group. To this end the absolute difference between the “standard rating” and the rating made by each participant will be calculated.
2. It is also expected that the scored overall quality will be lower for the intervention group since studies have found that people using explicit criteria as a base for evaluation tend to be more critical .

Both hypotheses will be tested using one-sided T-tests.

- Satisfaction with the portal

Satisfaction scales measuring the domains on 1- 7. Each domain will be summarised using mean, standard deviation, median and Inter Quartile range.

-Self-efficacy, attitudes and perceived pressure associated with searching for information (the theory of planned behaviour)

For each of the sub-domians attitude, subjective norm and perceived behavioural control of TPB-questionnaire a (weighted) composite score(see attachment for further information) will be calculated. The direction of effect is expected to be positive, in that improvements will be seen in sub-domains perceived behavioral control and attitudes, but may also be seen in increased perceived pressure. This will be t-tested using T-tests (two-sided).

An item analysis (Crohnbach’s alpha) exploring internal consistency within each composite score relating to the direct measures will be conducted. The TPB manual estimates that an acceptable internal consistency co-efficients should be approximately >0.6.

In the main analysis, the dependency between “intention” and the composite scores from the TPB questionnaire will be investigated using a multiple regression model, with “intention” as the dependent variable. Group assignment (portal vs no intervention), the composite scores for each direct measure and interaction terms between group assignment and composite score will be entered as independent variables.

-The patient activation measure (PAM)

The patient activation measure includes 13-items and the instrument is scored from 0-100, where 100 indicate high activation and 0 no activation .

The mean difference in overall score will measured. The direction of effect is expected to be positive in that those who receive the information will improve their level of activation. This effect will be tested using a two-sided T-test.

**Ethical aspects**

All participants will be informed about the purpose of the study and will be asked to sign informed consent forms. Data will be treated anonymously, and ethical approval has been granted by the Norwegian Social Science Data Services (NSD) and Regional Committees for Medical and Health Research Ethics (REK).

**Trial management**

*Registering the trial*

The trial has been registered.

*Trial management*

The trial will be led and managed by Astrid Austvoll-Dahlgren, under the supervision of Professor Sølvi Helseth, Professor Arild Bjørndal with the support of senior researcher Andy Oxman and Signe Flottorp and statistician.

**Consumer involvement**

The intervention is a tailored intervention based on previous studies and literature searches, but has also been evaluated and subsequently revised based on extensive consumer input.

**Reporting, Dissemination and Notification of results**

The results will be published in an international journal.

**References**
